# Supplementary material for: Causal interplay between lactose intolerance and gut microbiota: a combined bidirectional Mendelian randomization and in vivo validation study
Source: Front Nutr. 2026 Jun 1;13:1803337. doi: 10.3389/fnut.2026.1803337 (PMC13265576; doi:10.3389/fnut.2026.1803337)
Supplement: Supplementary file 1 [file Data_Sheet_1.zip › supplementary materials/Forward/leave one out cross validation/ebi-a-GCST90027832.finngen_R12_E4_LACTONAS.pdf]

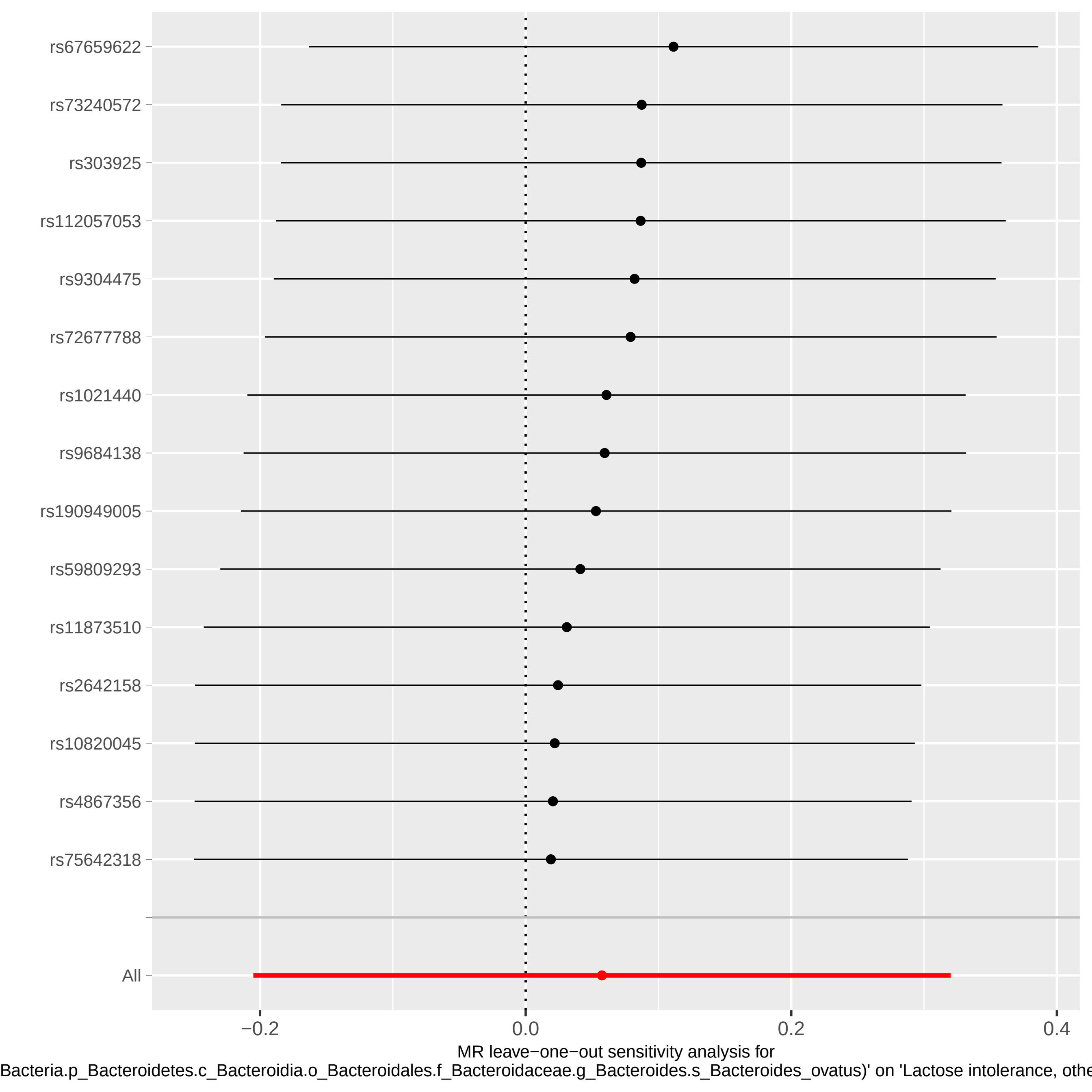
rs67659622
rs73240572
rs303925
rs112057053
rs9304475
rs72677788
rs1021440
rs9684138
rs190949005
rs59809293
rs11873510
rs2642158
rs10820045
rs4867356
rs75642318
All
−0.2
0.0
0.2
0.4
MR leave−one−out sensitivity analysis for
Bacteria.p_Bacteroidetes.c_Bacteroidia.o_Bacteroidales.f_Bacteroidaceae.g_Bacteroides.s_Bacteroides_ovatus)' on 'Lactose intolerance, othe
